# Fabrication of Spray-Dried Microcapsules Containing Noni Juice Using Blends of Maltodextrin and Gum Acacia: Physicochemical Properties of Powders and Bioaccessibility of Bioactives during In Vitro Digestion

**DOI:** 10.3390/foods9091316

**Published:** 2020-09-18

**Authors:** Chuang Zhang, Siew Lin Ada Khoo, Peter Swedlund, Yukiharu Ogawa, Yang Shan, Siew Young Quek

**Affiliations:** 1 Food Science, School of Chemical Sciences, The University of Auckland, Auckland 1010, New Zealand; zhangchuangchina@gmail.com (C.Z.); asie098@aucklanduni.ac.nz (S.L.A.K.); p.swedlund@auckland.ac.nz (P.S.); 2 Graduate School of Horticulture, Chiba University, 648, Matsudo, Matsudo 271-8510, Japan; ogwy@faculty.chiba-u.jp; 3 Hunan Key Lab of Fruits &Vegetables Storage, Processing, Quality and Safety, Hunan Agricultural Product Processing Institute, Hunan Academy of Agricultural Sciences, Changsha 410125, China; 4 Riddet Institute, Centre of Research Excellence for Food Research, Palmerston North 4474, New Zealand

**Keywords:** microencapsulation, spray-drying, *Morinda citrifolia* L., maltodextrin, gum acacia, bioaccessibility, noni juice

## Abstract

Microencapsulation of fermented noni juice (FNJ) into powder format could protect bioactive compounds, reduce the unpleasant odour and improve the acceptability for consumers. Blends of maltodextrin (MD) and gum acacia (GA) were used to achieve spray-drying microencapsulation of noni juice at different blending ratios. The physicochemical properties including microstructure, moisture content, water activity, particle size, bulk/tapped density, dissolution rate, ATR-FTIR and the bioaccessibility of bioactive compounds in powders during in vitro digestion were examined. Results showed that blends produced with more GA produced microcapsules with lower moisture content, water activity and bulk/tapped density, but slower powder dissolution. The ATR-FTIR results suggested that there were no significant chemical interactions between the core material and carrier or between the MD and GA in the blend powders. The spray-dried noni juice powder produced using the blends with higher ratio of GA to MD showed a better protection on the bioactive compounds, resulting in a higher bioaccessibility of powders during in vitro digestion. This study provides insights into microencapsulation of noni juice using blends of MD and GA and examines the physicochemical properties and bioaccessibilities of spray-dried powders as affected by the selected carriers.

## 1. Introduction

*Morinda citrifolia* L. (Noni) has been used for centuries as a medicinal plant, with reported therapeutic effects in the prevention or treatment of dyslipidaemia, diabetes, inflammation and cancer [1]. Approximately 200 bioactive compounds from the noni plant have been reported, including iridoids, anthraquinones, flavonoids, polysaccharides, glycosides, lignans and triterpenoids [2]. Iridoids are the major bioactive compounds in noni fruit, which have been demonstrated in a wide range of bioactivities in in vitro and in vivo studies, e.g., antioxidant, anti-inflammatory, antivirus, antibacterial and anti-arthritic [3]. Owing to the health benefits provided by noni fruit, the use of noni products as ingredients in foods has attracted people’s attention. However, noni juice has an unpleasant and pungent odour which limits customer acceptance.

Due to the potential health benefits of noni juice, it would be beneficial to transform noni juice into dehydrated products, such as powders, for stability, ease of transportation and handling, and to reduce the unpleasant odour. Dehydration would enhance the use of noni fruit as a functional ingredient in the formulation of a variety of health products and novel nutritional supplements. In addition, spray-drying microencapsulation of fruit juice is an efficient method used in preserving and delivering sensitive materials [4], and its application on noni juice has been studied [5,6]. These studies have examined the physicochemical properties (moisture content, particle size, tapped bulk density and hygroscopicity, etc.) and functionalities of noni juice microcapsules produced by systems with one hydrocolloid, either maltodextrin (MD, 10–13 and 17–20 DE (dextrose equivalent)) or gum acacia (GA), and elucidated the advantages of using MD (10–13 DE) or GA as carriers for microencapsulation. The purpose of this study is to investigate the performance of blended carriers combining both MD (10–13 DE) and GA for noni juice microencapsulation and to evaluate the impacts on in vitro digestion, to enhance the applicable possibility for industries.

Many studies have shown that both MD and GA have their own particular advantages and disadvantages in terms of cost, process efficiency and effect on the properties of spray-dried products [7,8]. For example, MD is a widely used carrier in industry due to its low cost and good solubility [5,9]. GA is considered to be an outstanding carrier for spray-drying microencapsulation, however, the high cost and limited supply are a disadvantage in the food industry [5,10]. Therefore, partial substitution of GA has been encouraged. In general, because each carrier has certain limitations, using a mixture of materials is often chosen in practical application. Studies have shown that blends of MD and GA can be more efficient than a single carrier in some systems [11,12].

The fact that noni juice has many potential health benefits means that it is important to understand and improve bioaccessibility of the bioactives during digestion. The bioavailability of polyphenols in the gut is reflected in the antioxidants, which are released from the food matrix. These antioxidant compounds penetrate through the cell membrane and play a role within the cell [13]. This can be quantified by evaluating the polyphenols during digestion, metabolism and absorption in the body [14,15]. A number of studies have tested in vitro digestion of fruit and vegetable to elucidate the changes of phenolics and antioxidant activity after digestion [16,17,18,19]. It is known that the complexity of the food system influences the release of certain compounds in ways that depend on their specific chemical structure. Antioxidant activity is susceptible to many parameters during digestion, in part because the polyphenol molecules are normally associated with carbohydrates, amino acids and other molecules which can affect the release of these associated compounds [20].

Microencapsulation of noni juice could reduce the undesirable odour and maximise their bioaccessibility for better acceptance and consumption of consumers [6,21]. This study aimed to examine the physicochemical properties, functionalities and bioaccessibilities of noni juice microcapsules affected by the blends of MD and GA, providing a fundamental knowledge to improve the industrial application on microencapsulation of noni juice.

## 2. Materials and Methods 

### 2.1. Materials and Reagents 

Fermented noni juice (FNJ) with ~7% solids was obtained from a health store (Auckland, New Zealand). Carriers including maltodextrin (MD: 10–13 DE) and gum acacia (GA) were kindly given by Ingredion Ltd. (Auckland, New Zealand). HPLC (High-Performance Liquid Chromatography)-grade acetonitrile was bought from JT Baker (Phillipsburg, NJ, USA), formic acid was purchased from Romil Ltd. (Cambridge, UK), Folin–Ciocalteus’s phenol reagent and acetic acid were purchased from Merck KGaA (Darmstadt, Germany) and 2,2-azino-biz-3-ethylbenzothiazoline-6-sulfonic acid (ABTS) diammonium salt was from Abcam (Cambridge, UK). Reagents for in vitro digestion including sodium acetate, 8x USP pancreatin from porcine pancreas (P7545), pepsin from porcine gastric mucosa (P7000) and bile salts (B8756) were purchased from Sigma-Aldrich (Auckland, New Zealand). Ammonium carbonate, magnesium chloride hexahydrate, potassium persulfate and sodium carbonate were from ECP Ltd. (Auckland, New Zealand), calcium chloride dehydrate (AR grade) was from Thermo Fisher Scientific (Auckland, New Zealand) and sodium hydrogen carbonate (AR grade) was obtained from VWR (Auckland, New Zealand). All water used was of Milli-Q grade.

### 2.2. Feed Solution Preparation

FNJ was first filtered two times using a filter paper. According to the advantages and disadvantages mentioned above, the MD and GA were selected as carriers for spray-drying, with the different blended ratios of 5:5, 7:3 and 9:1 (*w*/*w*), respectively. The obtained powder samples were referred to as P55, P73 and P91, according to the ratios of MD to GA. The carrier was directly mixed with FNJ to achieve a core material to carrier ratio of 1:2. The mixture was stirred by a magnetic stirrer until completely dissolved and then stored at 4 °C until further uses. The solid content of feed solution was determined by an Atago hand-held refractometer (Model: ATC-IE, Brix 0–32%, Bellevue, WA, USA). 

### 2.3. Spray-Drying Process

**Figure A1:**
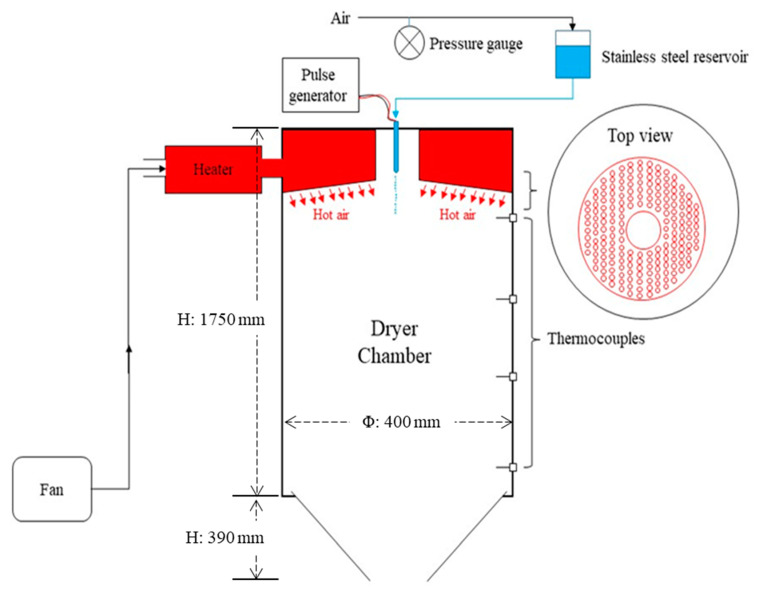
Schematic diagram of the micro-fluidic-jet-spray-dryer (MFJSD).

Spray-drying was conducted using a pilot-scale micro-fluidic-jet-spray-dryer (MFJSD) (Nantong Dong Concept Pty Ltd., Nantong, China) (Figure A1). The drying conditions were set up as follows: inlet air temperature 170 ± 2 °C, outlet air temperature 90 ± 2 °C, nozzle driver frequency 10 kHz, reservoir pressure 0.3 ± 0.05 kg/cm^3^ and the droplets were mono-dispersed at an air flow rate of 2 ± 0.5 L/min. Dispersion of the air was adjusted depending on the angle of the dispersed air (~40°). The feed solution was poured into a reservoir and was atomised into the spray-dryer using a microfluidic nozzle. The nozzle was made with a polytetrafluoroethylene (PTFE) tube and a ruby/sapphire orifice with a diameter of 75 μm. The spray-dried microcapsules produced were collected and transferred into a centrifuge tube. The sample tube was flushed with nitrogen gas and wrapped using a parafilm. The samples were stored in a desiccator containing silica gel in a refrigerator at 4 °C prior to further analysis.

### 2.4. Morphological Observation

The microstructures of FNJ microcapsules were observed by scanning electron microscope (SEM). Powders were fixed on an aluminium stub using a double-sided carbon tape and were sputter-coated with gold (DSR 1, Nanostructured Coating Co., Tehran, Iran). The observation was conducted by a benchtop SEM (TM3030 Plus, Hitachi Ltd., Tokyo, Japan) operated at 15 kV. The particle size of the microcapsule was analysed from the obtained SEM images by Image J software (US National Institutes of Health, Bethesda, MD, USA).

### 2.5. Moisture Content and Water Activity

The moisture content of the sample was measured according to the gravimetry method, drying in an oven at 105 °C until a constant weight was achieved. The water activity (*a*_w_) of samples was measured by a water activity meter (AQUALAB 4TE, Meter Group Inc., Pullman, WA, USA). The sample container was filled with powder sample to not more than 2/3 of the container capacity and placed in the chamber. The results are displayed on the screen once equilibrium is reached. 

### 2.6. Bulk Density and Tapped Density

Bulk density (*ρ*_bulk_) and tapped density (*ρ*_tap_) of the sample were measured according to Sarabandi et al. [7] with some modification. Briefly, sample (~2 g) was weighed and gently poured into a 10 mL dry graduated cylinder. The corresponding volume was noted to calculate the *ρ*_bulk_. The cylinder was then tapped 200 times to obtain the new powder volume. The *ρ*_bulk_ and *ρ*_tap_ were calculated according to Equations (1) and (2), respectively.

*ρ*_bulk_ = mass/volume _bulk_ (1)

*ρ*_tap_ = mass/volume _tap_ (2)

### 2.7. Dissolution Rate 

The dissolution rate of powder was determined according to Omobuwajo et al. [22] with some modifications. Briefly, powder (1 g) was dispersed in a 100 mL beaker containing 25 mL distilled water and stirred at 500 rpm. The time taken for the complete dissolution of samples was recorded. 

### 2.8. Glass Transition Temperature

The glass transition temperature (*T*_g_) of powder was determined by a Differential Scanning Calorimeter (model DSC 1000, TA Instruments, New Castle, PA, USA). Dry nitrogen was used as the purge gas at a flow rate of 25 mL/min, while indium and zinc were used for the calibration of temperature and heat flow. Spray-dried powder (7 to 8 mg) was weighed into a 25 µL aluminium pan which was then covered with a lid and sealed with a Tzero press. The samples were cooled down to −10 °C and then kept at −10 °C for 2 min before starting the scan. The scanning was carried out from −10 to 120 °C with a heating rate of 10 °C/min. The midpoint value for *T*_g_ was calculated using the TA universal analysis software (TA Instruments).

### 2.9. Attenuated Total Reflection-Fourier Transform Infrared Spectroscopy (ATR-FTIR)

The potential interaction between core material and carrier was investigated using the ATR-FTIR (Vertex 70, Bruker Pty Ltd., Billerica, MA, USA). Isopropyl alcohol was used to clean the diamond surface prior to the analysis. A small quantity of sample was placed on the diamond surface and clamped with a consistent low pressure. Infrared spectra were obtained against an air blank as the average of 16 scans with a 4 cm^−1^ resolution and measured between 600 and 4000 cm^−1^. Spectra were processed with a linear baseline between 800 and 1500 cm^−1^ and normalised to area.

### 2.10. Determination of Bioactive Compounds

#### 2.10.1. Total Phenolic Content (TPC)

The Folin–Ciocalteu (FC) reagent assay was used to measure the TPC of samples according to Tang et al. [23]. In brief, 25 µL of sample with an appropriate dilution was added into a 96-well plate. Then, 125 µL of 10-fold diluted FC reagent was added to the sample and followed by a reaction for 10 min. After that, 125 µL of sodium carbonate (7.5%, *w/v*) was added followed by an incubation of 60 min. The absorbance of the sample was determined at 765 nm by a UV/Vis microplate reader (EL 340, Bio-Tek Instruments Inc., Winooski, VT, USA). A standard curve was established through gallic acid water solutions with concentrations of 0, 0.05, 0.1, 0.2, 0.4 and 0.6 mM. The TPC value was expressed as mmol gallic acid equivalent (GAE) per litre of FNJ (mmol GAE/L). 

#### 2.10.2. Total Antioxidant Activity

The antioxidant activity of sample was determined by the ABTS assay according to Ozgen et al. [24] and Chen et al. [25] with modification. Briefly, the ABTS solution (7 mM) was prepared by 20 mM acetate buffer (pH~4.5) and then was mixed with potassium persulfate (2.45 mM) with the ratio of 1:1. The mixture was placed in a dark place for 12 to 16 h. Then, it was diluted using an acetate buffer to achieve the OD_734_ value of 0.70 ± 0.01. Ten µL of each sample with an appropriate dilution was added into a 96-well plate followed by a 190 µL of the ABTS^• +^ solution prepared previously. The mixture was left in the dark and allowed to react for 1 h prior to the OD_734_ value being measured by a microplate reader (EL 340, Bio-Tek Instruments Inc.). A standard curve was established by Trolox solutions (0, 0.05, 0.1, 0.2, 0.3 and 0.4 mM) prepared in ethanol. The antioxidant activity was expressed as mmol Trolox equivalent per litre of FNJ (mmol TE/L).

#### 2.10.3. Deacetylasperulosidic Acid (DAA) and Asperulosidic Acid (AA) Content

The iridoid (DAA and AA) content was determined using the HPLC method as described by Zhang et al. [4]. Chromatographic separation was done on a HPLC (HP 1100, Agilent Technologies, Wilmington, DE, USA), equipped with a ZORBAX Bonus-RP reverse phase column (4.6 × 250 mm, 5 μm) (Agilent Technologies). The mobile phase consisted of 0.1% (*v*/*v*) formic acid in water (mobile phase A) and acetonitrile (mobile phase B). The injection volume was 10 μL. The flow rate was set to 1 mL/min and the detector was monitored at 237 nm. The mobile phase was programmed as follows: 0–8 min, 98% A; 8–25 min, 98–85% A; 25–26 min, 85–10% A; 26–40 min, 10% A; 40–41 min, 10–98% A; 41–50 min, 98% A. The mixed standard solution containing DAA and AA with different concentrations of 0, 0.05, 0.1, 0.2, 0.3, 0.4 and 0.5 mg/mL were prepared in methanol, which were used to establish a standard curve for the calculation of DAA and AA content.

### 2.11. In Vitro Digestion Study

The in vitro digestibility of powder was examined according to Minekus et al. [26]. Electrolyte stock solutions were prepared as shown in Table 1.

To stimulate the oral phase, 0.2 g of sample was mixed with 140 µL of simulated salivary fluid (SSF), 30 µL of salivary α-amylase solution (1500 U/mL) and 1 µL of CaCl_2_ (0.3 M). Small amounts of HCl (1 M) were used to adjust the pH to 7 ± 0.5 followed by adding distilled water to obtain a final ratio of sample to SSF of 50:50 (*w/v*). The solution was incubated in a water bath (SWB20D, Ratek Instruments Pty Ltd., Boronia, Victoria, Australia) at 37 °C, 120 strokes/min for 2 min. To stimulate the gastric phase, the chyme (200 µL) from mouth phase was mixed with 150 µL of simulated gastric fluid (SGF) followed by 32 µL of pepsin solution (2000 U/mL) and 0.1 µL of 0.3 M CaCl_2_. A small amount of HCl (6 M) was added to adjust the pH to 3 ± 0.5 followed by adding distilled water to obtain a final ratio of sample to SGF of 50:50 (*v/v*). The mixture was then incubated at 37 °C, 120 strokes/min for 2 h. Digestion phase in upper part of small intestine was stimulated by mixing the gastric chyme (200 µL) with 110 µL of simulated intestinal fluid (SIF) stock solution, 50 µL of pancreatin solution (3 mg/mL), 25 µL of bile salt solution and 0.4 µL of CaCl_2_ (0.3 M). The pH was adjusted by adding NaOH (4 M) to 7 ± 0.5 and distilled water was added to obtain a final ratio of sample to SIF of 50:50 (*v/v*). Similarly, the mixture was then incubated at 37 °C, 120 strokes/min for 2 h.

In vitro digestion of each sample was performed in an individual tube for each time point, including initial time point, after simulated oral digestion for 2 min, after simulated gastric digestion for 1 h (G1), after simulated gastric digestion for 2 h (G2), after simulated intestinal digestion for 1 h (I1) and after simulated intestinal digestion for 2 h (I2). The collected samples at each time point were immediately centrifuged (8000× *g*, at 4 °C for 10 min), and the supernatants were used for further analyses, including total phenolic content (TPC), total antioxidant activity and iridoids (AA and DAA) content, as presented in Section 2.10.

### 2.12. Statistical Analysis

All analyses were done in triplicate. Means ± SD (standard deviation) are presented for each experiment. Statistical analysis was conducted using Microsoft Excel 2016 (Microsoft, Seattle, WA, USA) and SPSS Statistics 19 software (IBM, New York, NY, USA). Data were analysed using one-way analysis of variance (ANOVA) and Tukey’s test for pair comparison to test the differences between means. The significance level was determined at the 95% confidence limit (*p* < 0.05). Multivariate Curve Resolution Alternating Least Squares method was used to analyse the ATR-FTIR spectra. 

## 3. Results and Discussion

### 3.1. Microstructure of FNJ Microcapsules

**Figure 1:**
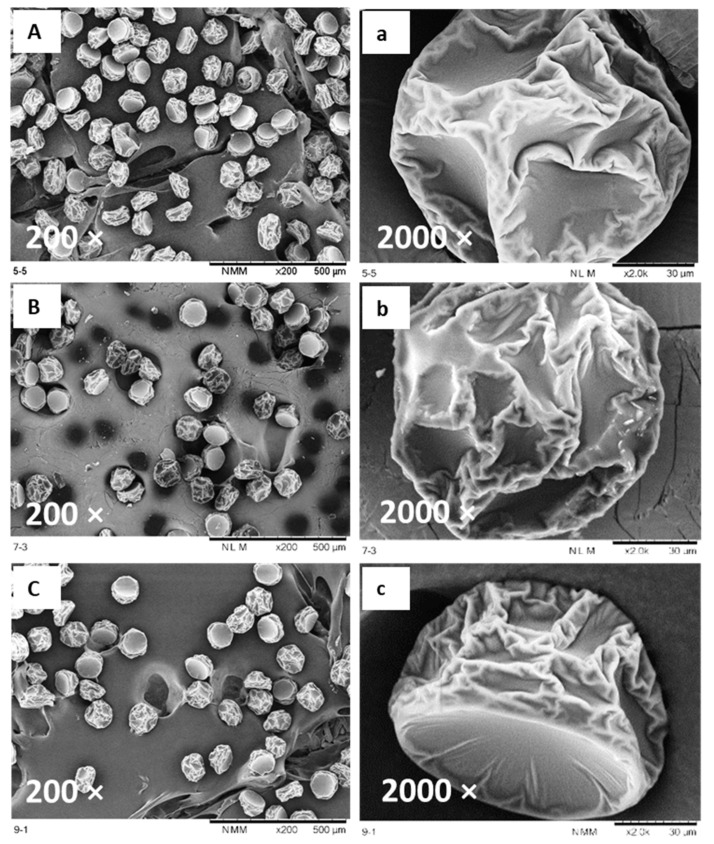
Scanning electron microscopy (SEM) micrographs of microcapsules ((**A**), (**a**): P55; (**B**), (**b**): P73; (**C**), (**c**): P91) at 200× and 2000× magnification.

All FNJ microcapsules exhibited a semi-spherical shape with relatively uniform size (Figure 1). This provided better control in powder properties and was more precise in the prediction of the performance for a specific application [27]. Figure 1 shows that the FNJ microcapsules had irregular surfaces with wrinkles. No obvious cracks were present in the samples which is a desirable feature as cracks would increase the exposed surface area and gas permeability which could affect the physicochemical/bioactive stability of the spray-dried powder. No significant difference on the degree of wrinkles of the microcapsules was observed. The occurrence of wrinkles is due to mechanical stresses caused by movement of moisture on the droplet surface during uneven drying [28]. This is consistent with the study on microencapsulation of grape polyphenol using MD and GA as carriers conducted by Tolun et al. [29].

### 3.2. Physicochemical Properties

#### 3.2.1. Particle Size Distribution

Particle size is related to storage properties, bioactive retention, handling and transportation of powders [30,31]. Generally, microcapsules with a smaller particle size have lower bioactive retention because of the larger surface exposed to the environment. In addition, properties including bulk density, solubility, hygroscopicity and flowability are strongly influenced by the particle size [9]. Powder particles produced by a conventional spray-dryer are often of broad size distribution due to the variety of sizes of the initial droplets after dispersion [32], implying different droplet drying patterns and rates. This may cause difficulty in the investigation of the structure and properties of an individual microcapsule formed by specific carrier. However, the FNJ microcapsules formed by the same feed solution using the micro-fluidic-jet-spray-dryer (MFJSD) showed a very narrow size distribution (Figure 1). 

**Figure 2:**
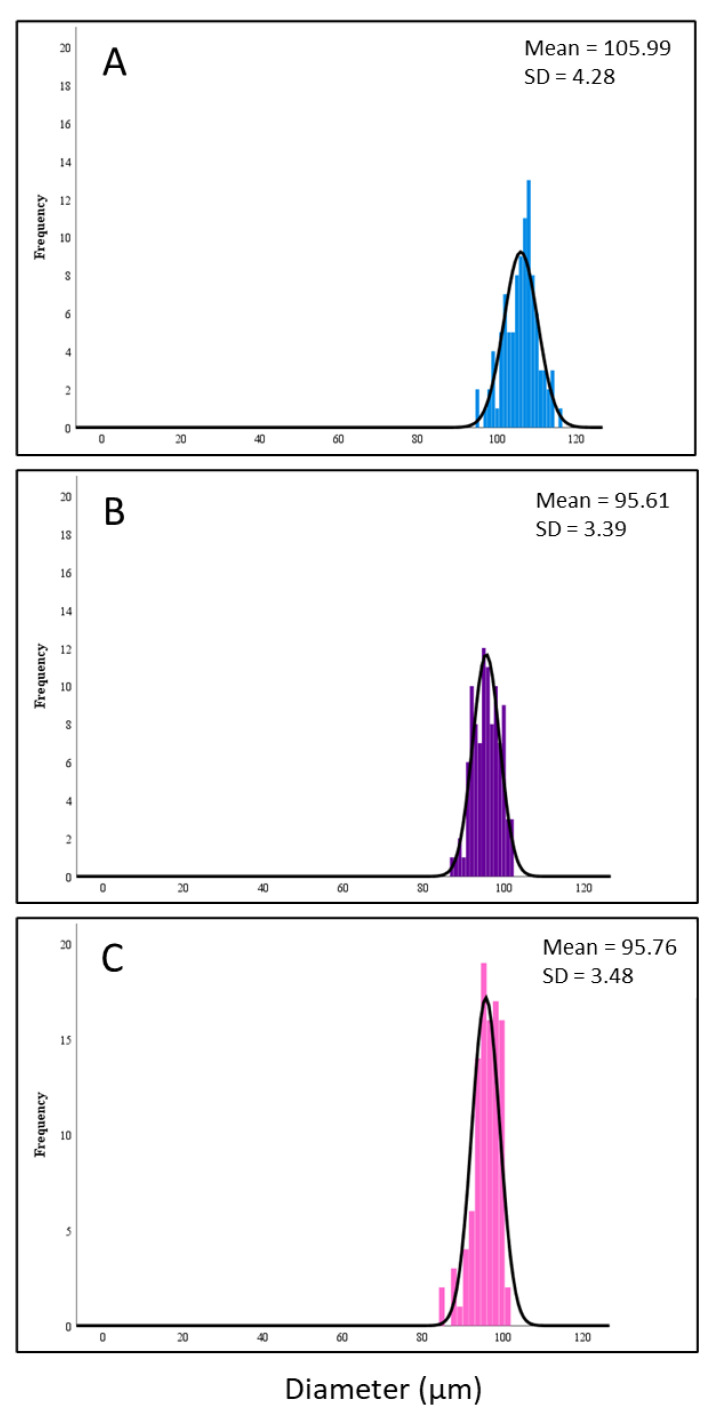
Particle size distribution of microcapsules: P55 (**A**), P73 (**B**) and P91 (**C**).

The mean particle sizes were 105.99, 95.61 and 95.76 µm for the powder with MD to GA ratio of 5:5, 7:3 and 9:1 (namely P55, P73 and P91), respectively (Figure 2). There was a significant difference (*p* < 0.05) between sample P55 and P73, while no significant difference (*p* ≥ 0.05) was observed between P73 and P91. The results indicated that the more GA used as a carrier, the larger particle size of microcapsule was obtained, which was consistent with previous studies [7,33]. GA consists of high molecular weight polysaccharides and its aqueous solutions exhibit a greater viscosity than that of MD [34]. The droplets formed during atomisation are greatly influenced by the viscosity of the feed solution [35]. A higher viscous of the feed solution could lead to a larger initial droplet size, resulting in a larger particle size of the microcapsule [36,37].

#### 3.2.2. Moisture Content and Water Activity

Moisture content is an important factor linked with the quality of spray-dried powder. Low moisture content can reduce the occurrence of powder caking, which is an undesirable phenomenon during powder storage [38]. The moisture contents of the FNJ powders varied from 5.17% to 5.57% with significant difference (*p* < 0.05) (Table 2), indicating that a higher MD to GA ratio of carriers led to a higher powder moisture content. This result was supported by previous studies of spray-dried juçara (*Euterpe edulis* M.) powder [39], lychee juice powder [40] and natural anthocyanins powder [41]. The effect could be due to the better moisture binding property of MD than GA, resulting in a higher content of residual water in the spray-dried powder.

Following the trend of the moisture content (P55 < P73 < P91), the water activities (*a*_w_) of the samples ranged from 0.15 to 0.22, with significant differences (*p* < 0.05), as shown in Table 2. A low *a*_w_ of a powder is favoured where enzymatic activity can be inhibited, and a reduced risk of microbial growth [30], with *a*_w_ being an essential index for spray-dried powder microbial stability. The shelf-life of spray-dried powder could be shorter if it has a high *a*_w_ due to the increasing amount of free water available for deteriorative reaction [42]. Since the *a*_w_ of all the FNJ powders were <0.3, they could be regarded as both microbiologically and chemically stable [30]. 

#### 3.2.3. Bulk and Tapped Density

Bulk and tapped density are essential factors for transportation and packaging of powders [43]. In this regard, a higher bulk density is desirable as it can reduce transport and packaging costs. The bulk densities of FNJ powders were 0.51, 0.56 and 0.58 g/mL for samples P55, P73 and P91, while those of tapped densities were 0.63, 0.69 and 0.69 g/mL, respectively (Table 2). Both bulk and tapped density were significantly (*p* < 0.05) influenced by the MD to GA ratio, following the tread: P55 < P73 < P91. Similar results were observed by Yousefi et al. [44], where lower bulk density was measured for spray-dried pomegranate juice powder produced with GA compared to those produced with MD. This tendency can be explained by the difference in particle size of microcapsules produced by MD and GA (refer to Section 3.2.1), resulting in different powder bulk and tapped densities. On the other hand, powder with higher bulk density could be due to the lower viscosity of feed solution, leading to a smaller particle size [45].

#### 3.2.4. Dissolution Rate 

The dissolution rate is an important criterion that is related to the sensory properties of reconstituted powders, which will directly affect the acceptability and marketability of powder products. The results (Table 2) indicated that an increased content of MD in the carrier improved powder solubility. This is supported by previous studies on spray-dried mango powder [46] and spray-dried lychee powder [40]. Two possible reasons could explain this phenomenon. As mentioned in Section 3.2.1, the higher viscosity of GA could result in a slower dissolution rate in water compared to MD. On the other hand, the smaller particle size of FNJ microcapsules produced with more MD may lead to a larger surface area to volume ratio exposed to the water, which would contribute to the faster dissolution rate and affect the reconstitution properties of powder. In addition, comparing the dissolution time (257 s) of spray-dried FNJ powder produced with GA only [5], the FNJ powder with MD to GA ratio of 5:5 obviously had a reduced dissolution time. This means the presence of MD as a carrier used in spray-drying microencapsulation of FNJ could effectively enhance the sensory evaluation of reconstituted powders. 

#### 3.2.5. Glass Transition Temperature

Glass transition temperature (*T*_g_) can be defined as the temperature at which the hard, solid, amorphous system changes its properties from a glassy state to a soft, rubbery state [47]. It is one of the important properties for powder stability as it has a strong influence on molecular mobility. When powder is stored below its *T*_g_, the mobility of molecules is hindered as the viscosity is greatly increased. On the other hand, caking and stickiness of powders are linked with the increase of molecular mobility when powders are stored above the *T*_g_ [48].

**Figure 3:**
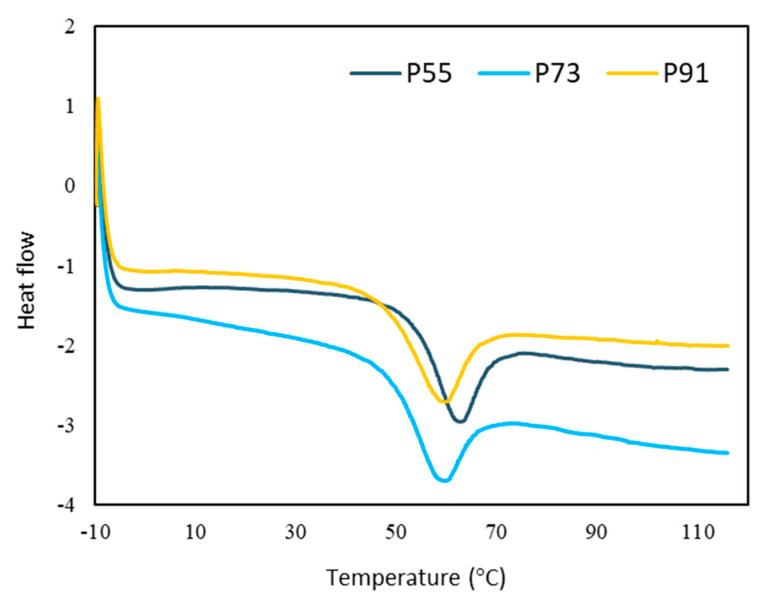
Glass transition temperature (*T*_g_) of spray-dried noni juice powders. P55, P73 and P91: powders produced using maltodextrin and gum acacia as carriers with the blended ratios of 5:5, 7:3 and 9:1.

Figure 3 showed that *T*_g_, the midpoint of the glass transition range, varied from 52.50 to 57.56 °C for samples P91 and P55, respectively. A significant difference (*p* < 0.05) between samples P55 and P91 was observed, and no significant difference (*p* ≥ 0.05) between samples P73 and P91. The Differential Scanning Calorimetry (DSC) thermogram indicated that sample P55, which contains the highest concentration of GA, has the highest *T*_g._ GA has greater molar mass compared to MD and therefore, GA increased the *T*_g_ of the powder. Truong et al. [49] suggested that molar mass has a positive influence on *T*_g_ and a similar phenomenon was reported for spray-dried lychee powder [40].

#### 3.2.6. ATR-FTIR Characterisation

**Figure 4:**
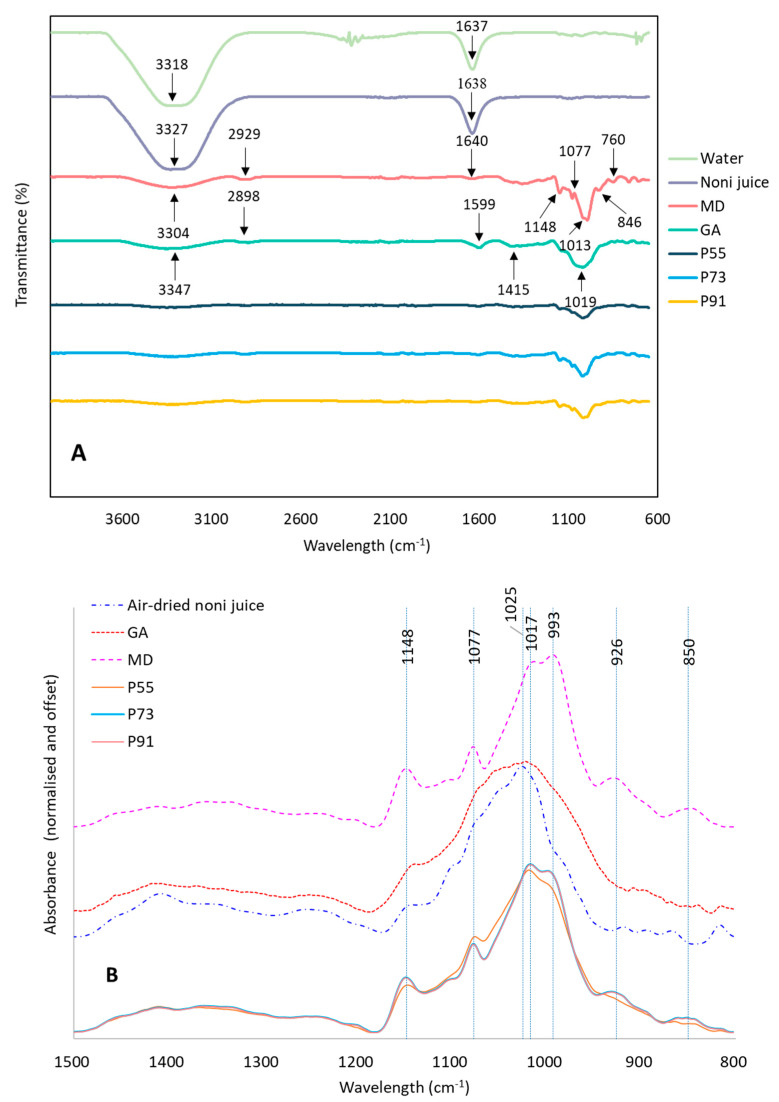
Attenuated Total Reflection-Fourier Transform Infrared Spectroscopy (ATR-FTIR) spectra of (**A**) Transmission spectra of water, pure noni juice, individual carrier (MD and GA) and spray-dried noni juice powders. (**B**) Absorption spectra of air-dried noni juice sample, individual carrier (MD and GA) and spray-dried noni juice powders. MD: maltodextrin; GA: gum acacia; P55, P73 and P91: powders produced using maltodextrin and gum acacia as carriers with the blended ratios of 5:5, 7:3 and 9:1.

FTIR analysis was carried out to investigate the presence of different organic functional groups and to probe the possible interactions between core material and carrier after spray-drying. Figure 4 shows that the bands from 3000 to 3700 cm^−1^ in the dry samples were related to hydrogen bonds and O-H stretching in carbohydrates, carboxylic acids and residual water [50]. Similarly, the peaks at ~1640 cm^−1^ were assigned to H-O-H bending and can be linked to the presence of water [51]. The peaks in the range from 2850 to 3000 cm^−1^ were related to the asymmetric and symmetric stretching of the C-H bonds [50]. The bands at 1148, 1077, 1013 and 1019 cm^−1^ were produced by C-O stretching, while the peaks at 846 and 760 cm^−1^ were assigned to C-H bending and ring puckering [52]. The peak at 1599 cm^−1^ of the GA sample is likely to include bands relating the H-O-H bending of residual water and the amide I and II bands, which occur at frequencies between 1540 and 1690 cm^−1^ [50], as GA contains a small amount of protein. The C=O stretching vibrations of the peptide bonds will result in the amide I band and C-N stretching vibration in combination with the N-H bending-produced amide II band [53]. On the other hand, the presence of the carboxylic group (COOH) in GA produced the peak at 1415 cm^−1^, this O-H in-plane bending band could be assigned to the COOH groups in the uronic acid residues of gum polysaccharides [54].

Possible interactions between FNJ and carriers were probed by comparing the infrared spectra of air-dried noni juice, carriers and the spray-dried powder samples (Figure 4A,B). The spectral region from 850 to 1500 cm^−1^ was studied as it is not influenced by the amount of water remaining in the sample (Figure 4B). There were no new bands in the spray-dried juice powder with all the features being evident in the component spectra. Furthermore, the spectra in this region were analysed using MATLAB^®^ according to the Multivariate Curve Resolution Alternating Least Squares method [55]. Results showed that the IR spectra of the powders could be very closely described (99.98% of the variance) by using a linear combination of the spectra measured of the pure ingredients (MD, GA and air-dried noni juice). No distinct new features were developed in the IR spectra, suggesting no direct significant chemical interaction between the ingredients. This may be good since the bioactive compound may still maintain their structure, and thus, their activity, which could contribute to the bioactive stability of powder during digestion or storage.

### 3.3. Bioactive Stability during In Vitro Digestion 

To evaluate the bioactive stability of the powders and the protective effects of carriers during digestion, the simulated in vitro oral-gastric-intestinal digestion of FNJ powder was examined. The degradation of the total phenolic content (TPC), total antioxidant activity (TAC) and iridoids, including deacetylasperulosidic acid (DAA) and asperulosidic acid (AA), were investigated in the present study by measuring the percentage of retention after simulated mouth, gastric and intestinal digestion.

**Figure 5:**
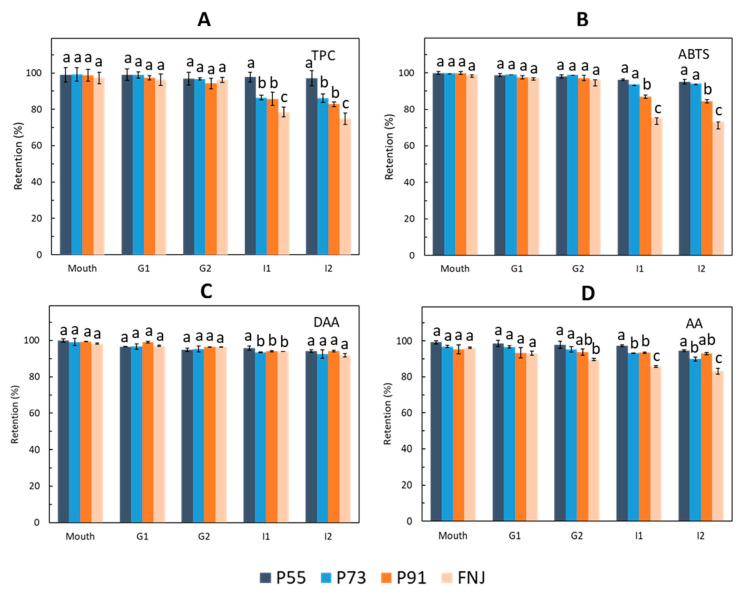
Retentions of TPC (**A**), antioxidant capacity (**B**) and iridoids contents (**C**, **D**) in spray-dried powders at different stages during in vitro digestion. DAA: deacetylasperulosidic acid; AA: asperulosidic acid; G1 and G2: after simulated gastric digestion for 1 and 2 h; I1 and I2: after simulated intestinal digestion for 1 and 2 h; P55, P73 and P91: powders produced using maltodextrin and gum acacia as carriers with the blended ratios of 5:5, 7:3 and 9:1. FNJ: fermented noni juice. The bars with different letters at the same in vitro digestion stage are significantly different (*p* < 0.05).

As shown in Figure 5A, the TPC in all spray-dried powders and the FNJ were highly retained (>94%) throughout mouth and gastric digestion. However, dramatic changes were observed after intestinal digestion for all spray-dried powder and FNJ, except for P55. The TPC in P73 and P91 dropped to 82–86%, and the lowest retention of TPC was found in FNJ (~74%). Notably, the P55 still retained over 97% in TPC, which means that TPC in the P55 was highly protected throughout the in vitro digestion. A similar phenomenon was found in the retention of antioxidant activity of samples during in vitro digestion (Figure 5B), which may be due to the contribution of polyphenol on antioxidant capacity. The dramatic degradation of phenolics in samples during intestinal digestion could be due to the high pH and the instability of the smaller molecules after being hydrolysed [56]. The iridoids (DAA and AA) in powder samples showed higher retentions than that in the FNJ throughout the simulated mouth, gastric and intestinal digestion (Figure 5C,D). Sample P55 showed the highest retention of DAA content (>94%), followed by P91 and P73. Similarly, AA content in the spray-dried powders had gradually decreased to 89–94% after simulated intestinal digestion. Results showed that the powder samples containing carriers had better bioactive accessibility than the FNJ during simulated digestion, indicating that blended MD and GA used as carrier could give protection for bioactive compounds of FNJ. On the other hand, the increased GA content of the blended carriers showed a higher bioactive retention, which implied that the GA could make more contributions than the MD for bioactive protection of powders during the digestion process. GA consists of a complex heteropolysaccharide with a highly ramified structure. It contains a small amount of protein linked to the carbohydrate chain, serving as a good film-forming agent. This provides a remarkable entrapping efficiency of the microencapsulated molecules. In this case, therefore, it makes the bioactive compounds less susceptible to degradation during digestion. The results found in the present study are supported by the investigations on microencapsulation of turmeric oleoresin [57] and spray-dried roselle (*Hibiscus sabdariffa* L.) extract using similar carriers [58]. 

In this study, α-amylase was used for oral digestion in order to mimic the presence of a salivary enzyme in the mouth. α-amylase is a digestive enzyme that can hydrolyse large polysaccharides including starch and glycogen into smaller by-products such as glucose and maltose by cleaving the α-1,4 glycosidic linkages [59]. The optimum pH for salivary amylase is 6.8 [60]. Despite the fact that food often has a short exposure time in the mouth, this salivary enzyme is essential as gastric acid will inactivate α-amylase, and almost no chemical breakdown of polysaccharides occurs in the stomach. This could explain why the desirable bioactivities of spray-dried powders were maintained after simulated gastric digestion (Figure 5A–D). MD is a mixture of saccharides, oligosaccharides and polysaccharides, and digestion of MD mainly occurred in the mouth and intestinal phase. However, mouth digestion was conducted for 2 min in the present study and therefore, the short retention time in the mouth may explain the high but reduced retention of bioactive compounds. In addition, amylase also presents in the intestinal phase, and this phase was conducted for 2 h. Thus, the polysaccharide can have a further breakdown in the intestinal phase, causing a dramatic reduction of bioactive components in spray-dried powder. A similar result was observed in a previous study [61].

The results approved that the phenolics and iridoids in FNJ could be mainly degraded upon in vitro intestinal digestion. However, microencapsulating FNJ into powder form could enhance the bioaccessibility of these bioactive compounds during in vitro digestion. In addition, a suitable carrier selection and blending proportion could improve the applicability of microencapsulation in industry. 

## 4. Conclusions

This work investigated the spray-drying microencapsulation of noni juice using blends of maltodextrin (MD) and gum acacia (GA) as carriers, looking at the physicochemical properties and bioaccessibility of powders as affected by the blending proportions. Overall, results showed that an increased ratio of GA to MD could lead to a better powder flowability and stability, while the more MD used in the blends could result in a better powder reconstituted rate in water. No significant interactions between noni juice and carrier, and different carriers, were observed according to the ATR-FTIR analysis. A higher blending ratio of GA to MD could give a better protection on the bioactive compounds in noni juice powders during simulated in vitro digestion, resulting in a higher bioaccessbility. For further work, spray-drying noni juice using other carriers such as whey protein isolate, starch and chitosan can be conducted to investigate the protection on bioactive compounds during drying and in vitro digestion.

## Figures and Tables

**Table 1:** Preparation of the simulated fluid stock solution.

	Stock Concentration	SSF, pH 7	SGF, pH 3	SIF, pH 7
Constituent			Concentration	
	mol/100 mL	mmol/100 mL	mmol/100 mL	mmol/100 mL
KCl	0.05	1.51	0.69	0.68
KH_2_PO_4_	0.05	0.37	0.09	0.08
NaHCO_3_	0.1	1.36	2.5	8.5
NaCl	0.2	-	4.72	3.84
MgCl_2_(H2O)_6_	0.015	0.015	0.01	0.033
(NH_4_)_2_CO_3_	0.05	0.006	0.05	-

SSF: simulated salivary fluid; SGF: simulated gastric fluid; SIF: simulated intestinal fluid.

**Table 2:** Physicochemical properties of spray-dried noni juice powders.

Properties	P55	P73	P91
Moisture content (%)	5.17 ± 0.05 ^a^	5.25 ± 0.08 ^a^	5.57 ± 0.14 ^b^
*a*_w_	0.15 ± 0.01 ^a^	0.18 ± 0.03 ^ab^	0.22 ± 0.04 ^b^
*ρ*_b_ (g/mL)	0.507 ± 0.025 ^a^	0.555 ± 0.005 ^ab^	0.583 ± 0.005 ^b^
*ρ*_t_ (g/mL)	0.628 ± 0.018 ^a^	0.689 ± 0.014 ^b^	0.689 ± 0.001 ^b^
Dissolution time (s)	47.5 ± 3.5 ^a^	36.5 ± 2.1 ^b^	25.5 ± 0.7 ^c^

*a*_w_: water activity; *ρ*_b_: bulk density; *ρ*_t_: tapped density; P55/P73/P91: powders produced with maltodextrin to gum acacia ratio of 5:5, 7:3 and 9:1. Values are presented as mean ± standard deviation. Different letters in the same row represent significant difference (*p* < 0.05).
